# On the Mechanism of Microwave Flash Sintering of Ceramics

**DOI:** 10.3390/ma9080684

**Published:** 2016-08-11

**Authors:** Yury V. Bykov, Sergei V. Egorov, Anatoly G. Eremeev, Vladislav V. Kholoptsev, Ivan V. Plotnikov, Kirill I. Rybakov, Andrei A. Sorokin

**Affiliations:** 1 Institute of Applied Physics, Russian Academy of Sciences, Nizhny Novgorod 603950, Russia; byk@appl.sci-nnov.ru (Y.V.B.); egr@appl.sci-nnov.ru (S.V.E.); aeremeev@appl.sci-nnov.ru (A.G.E.); holo@appl.sci-nnov.ru (V.V.K.); ivanplotnikov@appl.sci-nnov.ru (I.V.P.); asorok@appl.sci-nnov.ru (A.A.S.); 2 Advanced School of General and Applied Physics, Lobachevsky State University of Nizhny Novgorod, Nizhny Novgorod 603950, Russia

**Keywords:** microwave sintering, flash sintering, oxide ceramics, electric conductivity, grain boundary melting, densification, 81.40.Wx

## Abstract

The results of a study of ultra-rapid (flash) sintering of oxide ceramic materials under microwave heating with high absorbed power per unit volume of material (10–500 W/cm^3^) are presented. Ceramic samples of various compositions—Al_2_O_3_; Y_2_O_3_; MgAl_2_O_4_; and Yb(LaO)_2_O_3_—were sintered using a 24 GHz gyrotron system to a density above 0.98–0.99 of the theoretical value in 0.5–5 min without isothermal hold. An analysis of the experimental data (microwave power; heating and cooling rates) along with microstructure characterization provided an insight into the mechanism of flash sintering. Flash sintering occurs when the processing conditions—including the temperature of the sample; the properties of thermal insulation; and the intensity of microwave radiation—facilitate the development of thermal runaway due to an Arrhenius-type dependency of the material’s effective conductivity on temperature. The proper control over the thermal runaway effect is provided by fast regulation of the microwave power. The elevated concentration of defects and impurities in the boundary regions of the grains leads to localized preferential absorption of microwave radiation and results in grain boundary softening/pre-melting. The rapid densification of the granular medium with a reduced viscosity of the grain boundary phase occurs via rotation and sliding of the grains which accommodate their shape due to fast diffusion mass transport through the (quasi-)liquid phase. The same mechanism based on a thermal runaway under volumetric heating can be relevant for the effect of flash sintering of various oxide ceramics under a dc/ac voltage applied to the sample.

## 1. Introduction

In recent years considerable interest has been drawn to the processes of materials sintering making use of electric currents and/or fields. Enhanced sintering of various ceramic, composite and metal powder materials has been observed when using such methods as Field Assisted Sintering Techniques (FAST), Pulsed Electric Current Sintering (PECS), Spark Plasma Sintering (SPS), and Microwave Sintering. Several reviews of these methods and their applications to the sintering of a wide range of different ceramics have been published recently (see, e.g., [1,2,3]). These new techniques have attracted great attention due to their common advantage, viz. a shorter time needed to consolidate powders as compared to conventional methods. In many cases, the reduction in the sintering time can be as large as 10^2^, and the total processing time can be minutes instead of hours.

Recently, an even faster sintering method has been developed, the so called flash sintering [4,5,6,7,8]. In this method, a DC or low-frequency AC voltage is applied to a ceramic powder compact heated in a conventional furnace. The flash sintering occurs at a certain critical combination of the values of the temperature and the power dissipated in the sample due to the flow of electric current through it, and it results in full densification of the compact in a few seconds. The mechanisms responsible for flash sintering have not yet been determined, being a subject of wide discussion. Several hypothetic concepts supposedly relevant to flash sintering have been listed in the review [2]. They include nucleation of Frenkel pairs under the applied field [9], electrical boundary resistance [10], stress-induced generation of electric fields [11], and interaction between the external field and the space charge field [12]. Recently, a liquid film capillary model was discussed as a mechanism of rapid densification during flash sintering [13]. A similar mechanism of grain boundary softening has been proposed to explain the rapid densification under SPS [14]. In fact, starting from the very first publications on the flash sintering effect observations it has been noted that the current flow through the sample should lead to its significant heating [7]. The internal Joule heat sources provide volumetric heating, whereas the removal of heat proceeds through the surface. It is clear that at a certain ratio between the capacity of energy generation and heat capacity of the sample an overheating instability, known as the thermal runaway, may develop. Currently, it is generally agreed that the temperature instability plays a crucial role in the development of the flash sintering effect. At the same time, there is no clear understanding of how/whether the temperature instability can be a trigger for fast densification.

The thermal runaway is a well-known issue in microwave processing of materials [15,16]. Microwave heating occurs due to the absorption of electromagnetic radiation in the material. In many materials, including ceramics of various compositions, the coefficients of absorption increase with temperature. If during the heating process the parameter β = (*dP_v_*/*dT*) × (*T*/*P_v_*) (where *P_v_* is the power deposited per unit volume of the sample and *T* is temperature) exceeds a certain critical value, a thermal runaway develops in the sample [16]. As a rule, the possibility of uncontrolled temperature instability development is viewed as one of the main shortcomings of the use of microwave heating for high-temperature processing of materials.

We recently demonstrated [17,18] that flash sintering of oxide ceramic materials (Al_2_O_3_, Y_2_O_3_, MgAl_2_O_4_, and Yb:(LaY)_2_O_3_) is observed under rapid microwave heating due to the development of a temperature instability. There is experimental evidence that the ultra-rapid densification (within tens of seconds) of powder compacts to a near-theoretical density occurs due to particle surface softening and subsequent liquid phase sintering. In the development of the flash sintering process a determining role is played by two factors associated with the properties of the boundary regions of the grains. Due to the elevated concentration of defects and impurities in the grain boundaries these regions have higher electric conductivity (which grows fast as the temperature increases) and therefore higher absorption coefficient of microwave radiation. The increase in the absorbed power causes further increase in the temperature and in the effective electric conductivity. Although the power is deposited in a non-uniform manner because of a non-uniform distribution of the heat sources, thermal conduction effectively equalizes the temperature on the scale of the grain size (on the order of one micron or less) [19]. On the other hand, at temperatures that are close enough to the melting point the intense electromagnetic field may generate additional defects in the crystalline lattice. Due to the elevated defect/impurity concentration the temperature of the particle surface softening/pre-melting can be noticeably different from the melting point of the crystalline material of the bulk of the grains. Therefore it can be argued that intense microwave heating leads to formation of a (quasi-)liquid phase at the grain boundaries, which results in very rapid liquid-phase sintering. A proper fast feedback-based control of the microwave power during sintering helps prevent possible negative effects associated with the thermal runaway.

In this paper we discuss in detail the experimental results that confirm formation of the liquid phase in the samples sintered under intense microwave heating. The estimated values of the electric field, effective electric conductivity, and specific absorbed power are shown to be close to the respective values obtained in the flash sintering processes carried out under an applied DC or low-frequency AC electric field. Based on this correlation, we conclude that the mechanisms responsible for the flash sintering effect are similar for the DC/AC electric field-assisted processes and microwave sintering.

## 2. Materials and Methods

### 2.1. Materials Preparation and Characterization

Nanopowders of Y_2_O_3_, MgAl_2_O_4_, and 5 at % Yb:(La_0.1_Y_0.9_)_2_O_3_ compositions were produced at the Institute of Chemistry of High-Purity Substances (Russian Academy of Sciences, Nizhny Novgorod, Russia) via a wet chemical route with a subsequent self-propagating high-temperature synthesis (SHS). The details of the powder production procedures can be found elsewhere [17,18]. The particle sizes of powders after calcining determined by the Brunauer, Emmett and Teller method (BET) were 130 nm (Y_2_O_3_), 10 nm (MgAl_2_O_4_), and 140 nm (Yb:(LaY)_2_O_3_). The Al_2_O_3_ samples were prepared from high purity α-alumina powder AES-11C (Sumitomo Chemical Co. Ltd., Tokyo, Japan). The average particle size D_50_ was 0.45 µm. According to the producer, 0.05 wt % MgO was intentionally introduced into the powder for better sinterability. The powders were cold uniaxially pressed at 150–400 MPa into disks, 13 mm in diameter and 2.5 mm in thickness. The relative densities of the green bodies were approximately 0.42 (Y_2_O_3_), 0.38 (MgAl_2_O_4_), 0.52 (Yb:(LaY)_2_O_3_), and 0.64 (Al_2_O_3_) of the corresponding theoretical densities.

The samples were heated in the applicator of a gyrotron system with a microwave power of up to 6 kW at a frequency of 24 GHz, equipped with a computerized feedback power control circuit [20]. The samples were placed in the center of a cylindrical quartz crucible, 100 mm in diameter and 100 mm in height. For thermal insulation of the samples the crucible was filled with either coarse (particle size 3–5 μm) Y_2_O_3_ powder (in the case of Yb:(LaY)_2_O_3_ and Y_2_O_3_ samples) or coarse alumina powder (MgAl_2_O_4_ and Al_2_O_3_ samples). The temperature of the samples was measured by a B-type thermocouple whose tip touched the sample center from the bottom. In the case of Yb:(LaY)_2_O_3_ sintering, a thin (0.5 mm) Y_2_O_3_ ceramic disk was placed between the sample and the thermocouple to prevent chemical interaction. To remove the residues of the binder, samples were heated in air at a slow heating rate (10 °C/min) up to an intermediate temperature of 800 °C and held for one hour. No alterations in density and microstructure of samples were observed after this preliminary heat treatment. Upon completion of this initial stage of heating, the applicator was pumped out to a pressure of about 5 × 10^−1^ Pa and then the main stage of fast heating to the preset maximum temperature started. The Al_2_O_3_ powder compacts were microwave sintered in air at normal pressure.

The samples were heated from the above mentioned intermediate temperature to the maximum sintering temperature in two different regimes. One employed computer control of the microwave power enabling the heating of a sample at a preset fixed rate (50, 100, 150, or 200 °C/min). In the other regime, a fixed level of microwave power was used (about 5 kW as measured at the applicator input). In both cases the heating was terminated when the preset maximum temperature was reached. The microwave power was switched off automatically by the control system and the sample cooled down along with the thermal insulation surrounding it.

For the comparative study of the grain growth kinetics the samples were heated by microwaves and conventionally at a ramp-up rate of 6 °C/min to a number of temperatures in the range 1320–1770 °C and held at these temperatures for zero and ten hours. Mean linear intercepts, *L*, were measured on SEM images of thermally etched polished surfaces containing 50–150 grains, and converted to grain size, *D*, taking into account the stereological factor [21].

A key point in the comparative studies is the accordance between the results of temperature measurements at microwave and conventional heating. An unshielded B-type Pt–Rh thermocouple was used to measure the temperature of samples at microwave heating. The correctness of the thermocouple measurements was checked by microwave heating of a small copper ball making use of the Cu melting temperature (1083 °C) as a reference point. The results of tests have shown that the difference between the measured melting temperature and the reference data did not exceed 5 °C, which was within the accuracy of the thermocouple measurements.

The density of the sintered samples was determined by Archimedes weighing in distilled water with an estimated accuracy of ±0.01 g/cm^3^. The microstructure of the as-sintered samples was studied by scanning electron microscopy (JEOL JSM-6390 LV, Tokyo, Japan). The element distribution was analyzed by energy dispersion spectrometry (EDS) using JEOL EX-54175 JMH (Tokyo, Japan) detector combined with the microscope. The grain structure profiles were investigated by atomic force microscopy (Smena NT–MDT, Moscow, Russia). The phase composition of the sintered samples was analyzed using a Rigaku Ultima IV X-ray difractometer (Tokyo, Japan).

### 2.2. Energy Balance during Microwave Heating

As noted above, the connection between the development of the overheating instability (thermal runaway) and flash sintering is now commonly recognized. The instability develops due to a misbalance between the power deposited within the volume of the sample and the heat losses. Therefore a key parameter determining the flash sintering phenomenon is the power deposited per unit volume. While in the experiments with DC/low-frequency AC currents the power density is easily obtainable by measuring the current flowing through the sample and the applied voltage, in the case of microwave heating the power absorbed per unit volume is not readily available but can be determined using the procedure described below.

The microwave power absorbed in a sample increases its temperature and/or compensates the heat losses. The microwave power deposited per unit volume of the sample, *P_v_*, can be determined from the experimental data using the energy balance equations. Immediately before and after the time instant when the maximum temperature is achieved and the microwave power is switched off the energy balance equations take the following form:

(1) $$P_{v}=\rho C{\left(\frac{dT}{dt}\right)}_{+}+P_{hl},\;P_{hl}=\rho C\left|{\left(\frac{dT}{dt}\right)}_{-}\right|$$

where ρ is density, *C* is specific heat capacity of the material of the sample, *P_hl_* is the power in heat losses, (*dT*/*dt*)_+_ and |(*dT*/*dt*)_−_| are the rates of heating and cooling before and after the microwave power switchoff, respectively. As follows from Equation (1), the value of *P_v_* is higher, the higher the heating rate (as long as the thermal insulation conditions are the same). Therefore it can generally be argued that whenever the experimental results depend on the microwave heating rate, it is in fact the effect of the absorbed electromagnetic power.

**Figure 1:**
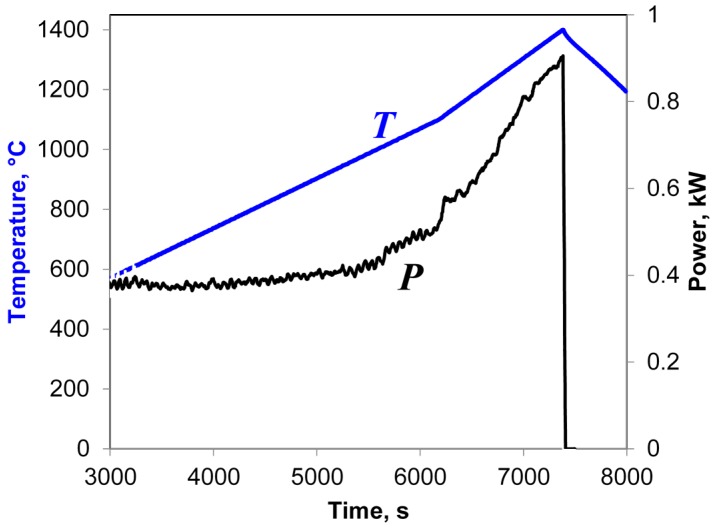
Temperature (T) and microwave power (P) during microwave heating of a sample compacted from α-Al_2_O_3_ powder at a rate of 10 °C/min to 1100 °C and then 15 °C/min to 1400 °C.

In general, the power in heat losses, *P_hl_*, is the total power lost by thermal conduction, convective and radiative heat flows. The power in heat losses increases as the temperature of the sample grows. Therefore, during a constant-rate heating process the microwave power deposited per unit volume of the sample increases monotonically to compensate the increasing heat losses (unless there are phase transformations accompanied with the changes in the internal energy of the sample). An example of such behavior of microwave power during the heating of a sample compacted from α-Al_2_O_3_ powder at a rate of 10 °C/min to 1100 °C and then 15 °C/min to 1400 °C is shown in Figure 1.

The power *P_v_* (1) absorbed in the sample, per unit volume, can be expressed via the electric field strength in the sample, *E_s_*, and the effective high-frequency electric conductivity of the material of the sample, σ_eff_, as follows:

*P_v_* = σ_eff_*E_s_*^2^ (2)

Unlike the experiments on flash sintering with dc/low-frequency ac currents, in the microwave sintering experiments it is not possible to directly measure the power absorbed in the volume of the sample. The measureable quantity in these experiments, along with the temperature of the sample, is the input microwave power, *Р*, that is fed into the applicator in which the sample and the thermal insulation arrangement surrounding it are positioned. The input power, *P*, determines the magnitude of the electromagnetic energy, *W*, stored in the cavity [22]:

(3) $$W=\varepsilon_{0}\int {Ε}^{2}dV=\frac{Q}{2\pi f}P$$

where ε_0_ is the electric constant, *E* is the electric field in the cavity, *Q* is the quality factor of the cavity, and *f* is the microwave frequency.

A specific feature of the gyrotron system that operates in the millimeter-wave range is that its applicator is an untuned multimode cavity [23] with a high ratio of its volume, *V*, to the cube of the radiation wavelength, λ: *V*/λ^3^ ≈ 10^5^. In such a cavity the electromagnetic field distribution over its volume is quasi-uniform since it presents a superposition of many oscillation modes excited simultaneously [24]. The quality factor, *Q*, for this type of cavity is determined primarily by the Ohmic losses in its walls, because the losses in the sample are small due to its small dimensions (usually on the order of 1 cm^3^) and the coupling loss is negligible due to small area of the input opening of the cavity.

It can be shown that the electric field strength *E_s_* in a small sample with the dielectric permittivity ε ~ 5–15, which is placed in the cavity, is approximately equal to the electric field in the cavity outside of the sample: *E_s_* ≈ *E*. Then, using Equations (2) and (3) it is easy to obtain the interrelation between the power incoming the applicator, *P*, and the microwave power deposited per unit volume of the sample:

(4) $$P_{v}\approx \frac{\sigma_{\text{eff}}}{2\pi \varepsilon_{0}f}Q\frac{P}{V}$$

where *V* is the volume of the applicator.

## 3. Results and Discussion

### 3.1. Flash Microwave Sintering of Yb:(LaY)_2_O_3_ Ceramic Samples

**Figure 2:**
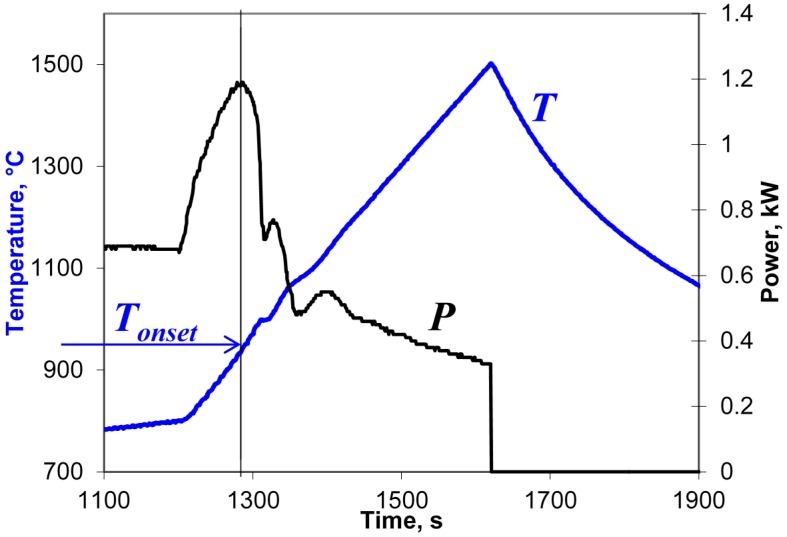
Temperature and microwave power at the applicator input vs. time, recorded at the high-temperature stage of the sintering of an Yb:(LaY)_2_O_3_ sample with a heating rate of 100 °C/min. The vertical line denotes the onset of the sharp rise in the effective conductivity.

In the present study, at temperatures above 800 °C the Yb:(LaY)_2_O_3_ samples were heated at rates in the range of 50–7000 °C/min up to a preset maximum temperature chosen between 1300 and 1600 °C. The cooling rates (immediately after the automatic microwave power switchoff at maximum temperature) were 180–1000 °C/min, depending on the heating regime. An example of a typical behavior of the input microwave power, *P*, during these microwave heating experiments is shown in Figure 2.

As seen in Figure 2, at an initial stage of heating the automatically controlled input microwave power increases from 680 to 1200 W as the temperature grows. Then, at a temperature of about 950 °C, the actual temperature growth rate begins to exceed the prescribed one. The accelerated temperature growth, i.e., thermal runaway, is suppressed by the automatic process control system by sharply reducing the microwave power. As follows from Equation (4), the drop in the power needed to sustain the heating at a constant rate means nothing but a sharp increase in the effective electric conductivity of the sample. The experiments show that the temperature *T_onset_* at which the power drop occurs decreases with increasing heating rate [17]. Obviously, the observed increase in the effective conductivity must be caused by certain changes in the structure or phase state of the material, which we will identify below. Let us note that the detection of such changes based on the measurement of the input microwave power can be viewed as a peculiar implementation of the method of microwave thermal analysis [25].

**Figure 3:**
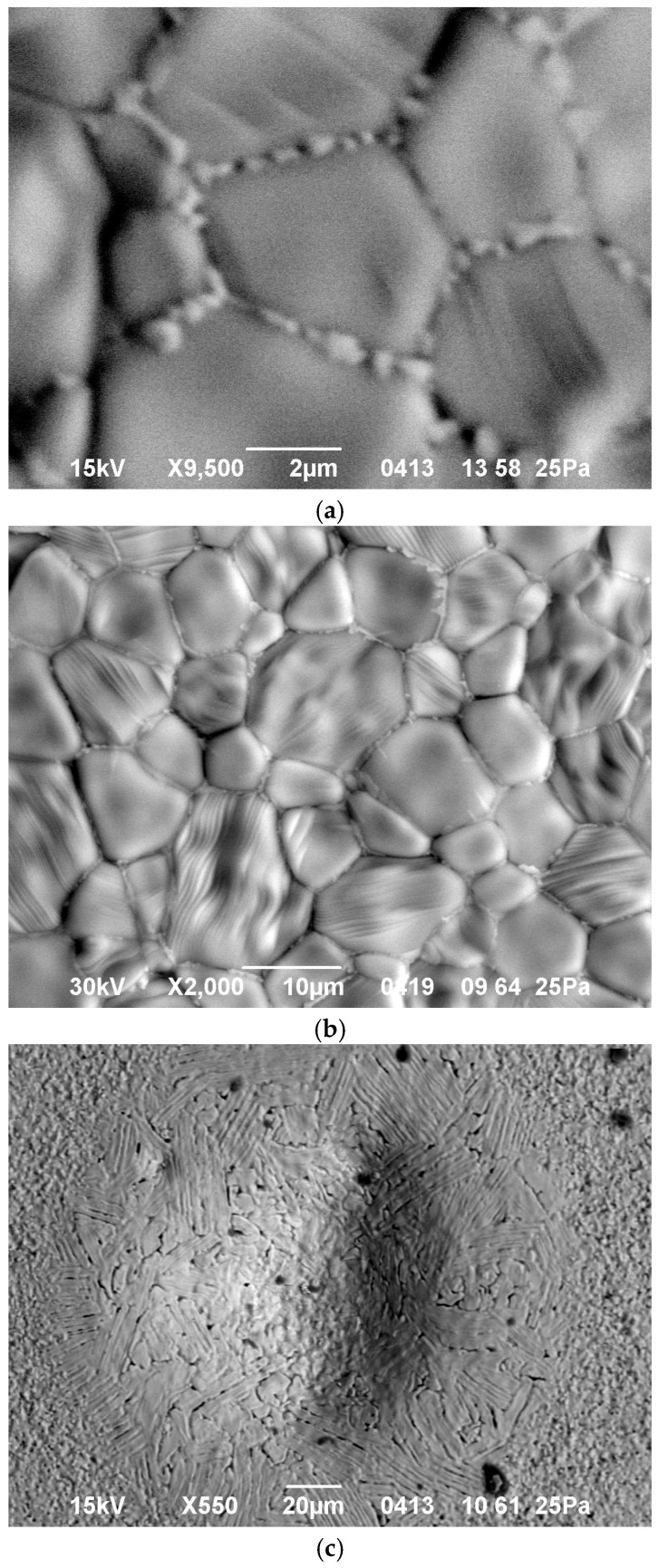
Microstructure of unpolished surfaces of sintered Yb:(LaY)_2_O_3_ samples heated in the regimes: (**a**) 50 °C/min to 1500 °C; (**b**) 100 °C/min to 1500 °C; (**c**) 7000 °C/min to 1580 °C.

The final density of all flash microwave sintered Yb:(LaY)_2_O_3_ samples was 98%–99% [17]. An SEM study of unpolished surfaces of the samples showed that the heating rate (and hence the absorbed microwave power as discussed above) strongly affects the microstructure. Isolated droplets, a fraction of a micron in size, located along the grain boundaries are observed in the sample heated at a rate of 50 °C/min to a temperature of 1500 °C (Figure 3a). In previous studies with samples of the same composition, microwave heated at a much slower rate of 5 °C/min [26], no such droplets were seen in the microstructure. With an increase in the microwave heating rate to 100 and 150 °C/min, the particles merge together forming layers, up to 0.3 µm in thickness, which surround the grains (Figure 3b). Between the grains one can clearly see interlayers composed of a different phase (looking brighter in the backscattered electron image). The formation of rounded or lenticular islands at grain boundaries that first produce a necklace structure and then aggregate into continued films is a well-known effect in the theory of liquid-phase sintering [27].

The thickness of the melted intergranular layers varies with the heating rate and the maximum temperature of the sample. If both the heating rate and the maximum temperature were excessively high, the melting process was not confined within the grain boundaries but affected larger (though still localized) areas of the sample (Figure 3c). An XRD study of the surfaces of all sintered samples, including those with partially melted regions, revealed only crystalline and no amorphous phases.

**Figure 4:**
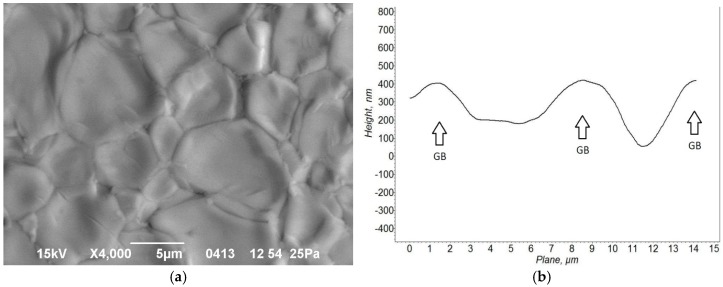
Microstructure of an unpolished surface of a sintered Yb:(LaY)_2_O_3_ sample microwave heated at a rate of 200 °C/min to 1500 °C: (**a**) SEM image; (**b**) AFM profile across two adjacent grains, grain boundary positions shown with arrows (note different scales for the horizontal and vertical axes).

The presence of a liquid phase between solid particles was confirmed by SEM and AFM images of the surfaces of the sintered ceramic samples. For example, an SEM study of the sample microwave heated at a rate of 200 °C/min to 1500 °C showed that the surface relief is uneven on the grain size scale (Figure 4a). The edges of the grains are raised above their middle area. The results of an AFM study of the surface relief confirmed that the grain edges are protruding (Figure 4b). Typically, the height of these edge ledges is of the same order of magnitude as the width of the softened intergranular layers, i.e., a fraction of a micron. The protruding areas arise at grain boundaries because the volume of material changes as the liquid phase forms. In most oxide materials the specific volume increases by 10%–20% upon transition from solid to liquid phase [28]. During sintering, the liquid phase partially fills the triple points of the granular structure by viscous flow, thus facilitating densification, and is partially extruded to the free surface of the samples.

The effect of accelerated sintering apparently results from an avalanche pre-melting or melting of powder particle surfaces [29,30]. The non-uniform deposition of microwave energy starts at an early stage of heating due to enhanced absorption of microwave energy at the particle surfaces, where the concentration of impurities and defects is elevated. Despite this non-uniformity in energy deposition, the temperature remains nearly uniform across the particle because thermal conductivity prevents development of a significant difference between the temperatures of the particle surface and bulk when the particle size is on the order of microns [19]. However, due to the abundance of impurities and defects in the near-boundary regions of particles the melting temperature of surface/boundary can differ noticeably from the melting point of the pure solid material. As a result, particle surface pre-melting may occur well below the bulk melting point of the material. This leads, in turn, to a sharp increase in the effective conductivity σ_eff_ and the absorbed microwave power *P_v_*, causing the development of a local thermal runaway. Due to the melting of particle surfaces the solid grains appear to be surrounded by a melt with a low viscosity. Dissolution of solid into the liquid and enhanced diffusion mass transport through the liquid layer leads to a rounded shape and a smooth surface of grains.

**Figure 5:**
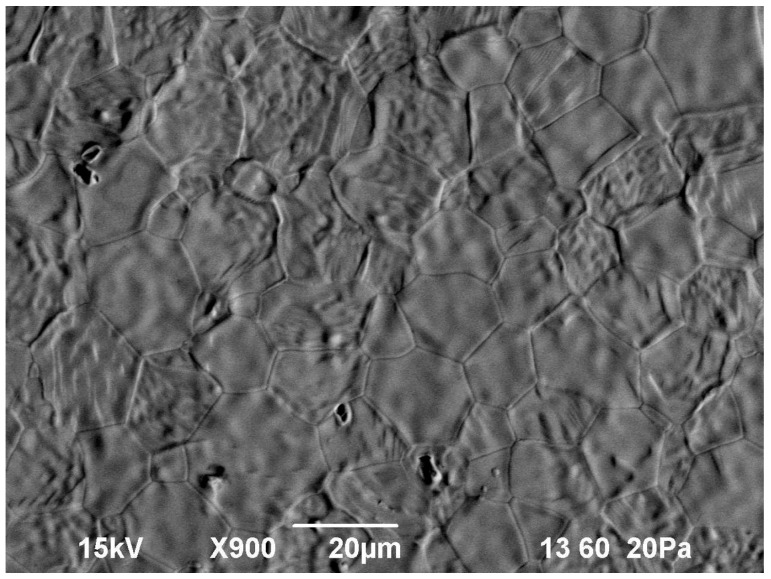
Microstructure of an unpolished surface of a sintered Yb:(LaY)_2_O_3_ sample microwave heated at a rate of 100 °C/min to 1570 °C. Note the substructure in some of the larger grains.

The liquid phase wets the grains completely because their chemical compositions are similar. The capillary attractive force causes particle rearrangement due to rotation and sliding of small-size grains relative to each other, which eventually results in fast densification. At the final stage of sintering the larger grains grow at the expense of the smaller grains by the solution-reprecipitation mechanism. As seen in the microstructure of an unpolished surface of a sample heated up to a temperature of 1570 °C (Figure 5), some of the larger-size (about 20 μm) grains have an inner substructure that consists of densely packed rounded particles of an order-of-magnitude smaller size. Note that a similar microstructure, with grains having an inner substructure, was observed in the YAG ceramics sintered by spark plasma sintering [31], which was interpreted as a manifestation of rapid densification of nanocrystalline YAG via surface softening of particles and liquid phase formation.

It is well known that during microwave volumetric heating, accompanied by heat loss through the surface, the so-called inverse temperature distribution develops in the sample, with the core of the sample being hotter than the periphery [16]. For example, during microwave heating at a rate of 150 °C/min to 1500 °C the temperature difference between the center and the surface of an Yb:(LaY)_2_O_3_ sample with a diameter of 13 mm reached 250–300 °C [17]. Yet, despite such a large temperature difference, the microwave flash sintered samples achieved uniform near-full density, and the grain size distribution over the diameter of the samples was fairly uniform, with deviations from the average value not exceeding ±10%. Based on the analysis of the experimental observations described above, the following mechanism of microwave flash sintering can be suggested. The process of particle surface melting starts in the core region of the sample. In the course of densification the liquid phase is partially squeezed out of the core region into the more porous peripheral structure. The region of the maximum deposition of the microwave power, *P_v_*, moves toward the periphery along with the liquid phase due to the elevated electric conductivity of the latter. This results in further melting of grain boundaries and liquid phase production outside of the core region of the sample. In this manner, a transient liquid-phase densification front propagates from the core of the sample to its periphery, resulting in high final density and uniform grain size distribution. It should also be noted that the liquid-phase front propagation limits the development of local thermal runaway at each particular point of the sample and thereby prevents its destruction.

**Figure 6:**
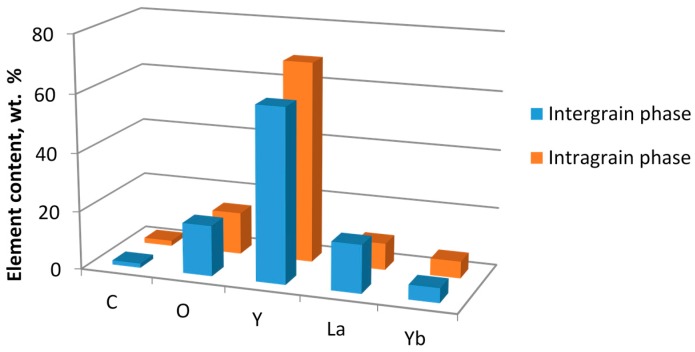
Results of element analysis on the surface of a sintered Yb:(LaY)_2_O_3_ sample microwave heated at a rate of 100 °C/min to 1550 °C.

An EDX study has shown that the initially uniform element distribution corresponding to the stoichiometric compound 5 at % Yb:(La_0.1_Y_0.9_)_2_O_3_ changes after rapid sintering. The characteristic element composition of the phases was determined by averaging the results of measurements at five points belonging to the intragrain areas and seven points in the intergranular phase. Each measurement characterized a spot on the order of 1 µm in size. The measurements were performed on unpolished surfaces of the samples to increase the sensitivity to the intergranular phase that squeezed out from between the grains and distributed partially over the surface of the sample. The results of element analysis, including the values of the experimental error, are listed in Table 1. A histogram of the element content in the intragrain and intergrain regions is shown in Figure 6.

Compared to the material of the grains, the intergranular phase is enriched with La and O and depleted with Y. This has also been confirmed qualitatively by element maps obtained by X-ray microanalysis. The relative element content in the bulk of the grains is close to the stoichiometric ratio for the composition 5 at % Yb^3+^ (La_0.1_Y_0.9_)_2_O_3_; however, in the intergranular phase it is notably different. Presumably, the La^3+^ ions have a lower diffusion coefficient than Y^3+^ due to their larger mass and ionic radius; therefore La ions may accumulate in the intergranular regions.

### 3.2. Microwave Effect on the Grain Growth Kinetics of Yb:(LaY)_2_O_3_ Ceramics

It is known that the Y_2_O_3_—(0–18 mol %) La_2_O_3_ solid solution with a cubic structure is thermodynamically stable at temperatures above 1400 °C [32,33,34]. No thermodynamic data on the 5 at % Yb^3+^ (La_0.1_Y_0.9_)_2_O_3_ composition, in particular on its melting temperature, are available. In order to study the effect of the microwave electromagnetic field on grain boundary melting phenomena, liquid phase formation, and its influence on grain growth, samples of this composition were heated both by microwaves and conventionally (in a resistive oven). This experimental series was performed at a ramp-up rate of 6 °C/min to make possible the comparison between the results of microwave and conventional heating.

**Figure 7:**
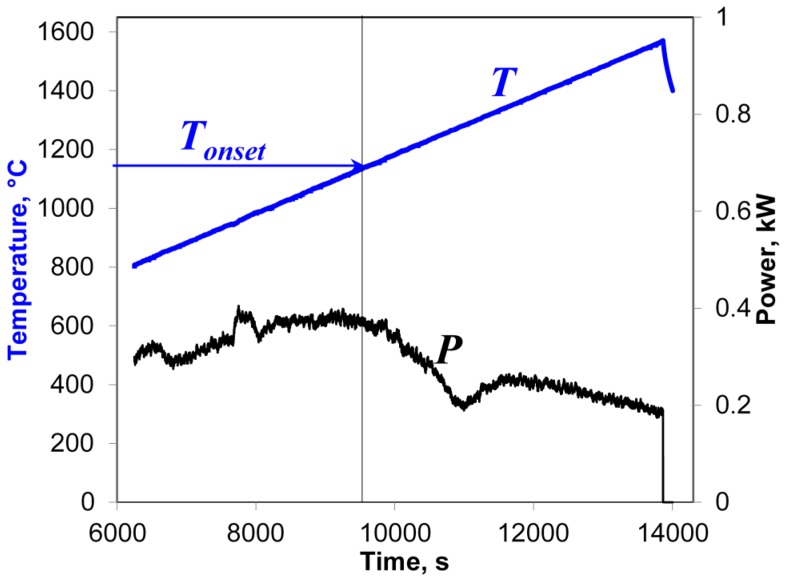
Temperature and microwave power at the applicator input vs. time, recorded at the high-temperature stage of the sintering of an Yb:(LaY)_2_O_3_ sample with a heating rate of 6 °C/min. The vertical line denotes the onset of the rise in the effective conductivity.

An example of a typical behavior of the input microwave power during the heating at a rate of 6 °C/min is shown in Figure 7. The vertical line denotes the onset of the rise in the effective conductivity presumably caused by the melting of particle surfaces. At this low heating rate, the onset temperature, *T_onset_*, is 1140 ± 20 °C, which coincides within the margin of error with the value 1130 ± 20 °C observed at a heating rate of 50 °C/min in the experimental series described in Section 3.1. This suggests that the temperature of particle surface softening in the presence of moderate-intensity microwave field is about 1140 °C, decreasing at higher field intensities.

It is well known that the process of grain growth is greatly accelerated in the presence of the liquid phase in the sintered material [35,36]. The rate of diffusion mass transport is proportional to the product of the diffusion coefficient and the cross section of the diffusion layer. At liquid phase sintering the magnitude of this product is high compared with the case of solid phase sintering due to an increase in both factors. As a result, the rates of both densification and grain growth are higher at liquid phase sintering. The features related to microwave radiation absorption and microwave interaction with the material may develop at any of the main consecutive stages of liquid-phase sintering [36]: (a) melting of the liquid-forming additive and redistribution of the liquid; (b) rearrangement of the majority solid phases in the presence of a liquid phase; (c) densification and shape accommodation of the solid phase; and (d) final densification driven by residual porosity in the liquid phase.

Therefore, a comparative study of the grain growth kinetics should make it possible to understand the mass transport mechanisms involved in the sintering process. The grain growth was systematically studied under microwave and conventional resistive heating by varying the temperature and hold time.

The grain growth by different mechanisms of diffusion mass transport is described by the following expression [35]:

*D^m^*(*t*) − *D^m^*(*t*_0_) = *Kt* (5)

where *D*(*t*) and *D*(*t_0_*) are the grain sizes at time *t* and *t*_0_, respectively, *m* is the grain size exponent,

*K* = *K*_0_ exp (−*Q_a_*/*RT*) (6)

*K*_0_ is the pre-exponential factor of the diffusion coefficient, *Q_a_* is the grain growth activation energy, and *R* is the gas constant. The exponent *m* depends on the rate-controlling mechanism of grain growth. The value *m* = 2 indicates that the solid state mass transport is the dominant mechanism for grain growth, whereas *m* = 3 is indicative of the diffusion though the liquid phase [37] or ion dissolution as the rate-controlling step.

**Figure 8:**
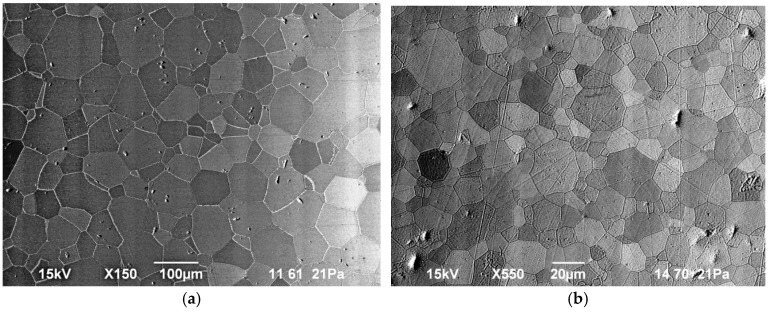
Microstructure of polished surfaces of sintered Yb:(LaY)_2_O_3_ samples heated at a rate of 6 °C/min to 1750 °C with a 10-h hold under (**a**) microwave, and (**b**) conventional heating. Note different scale bars.

**Figure 9:**
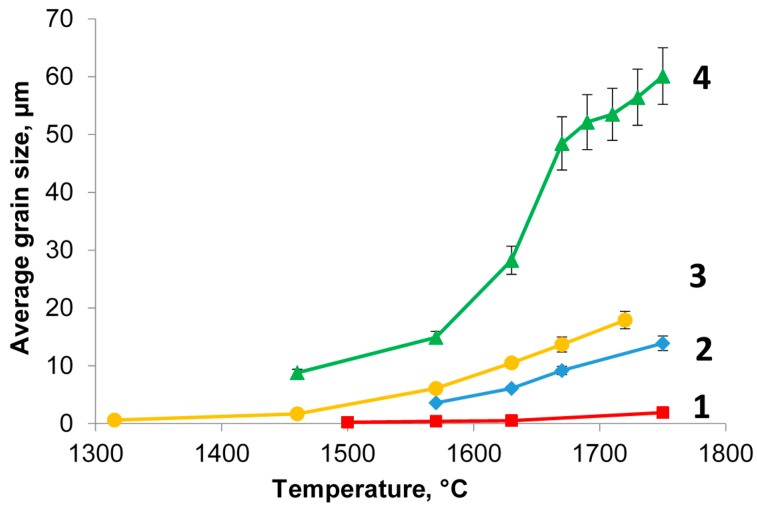
Average grain size in Yb:(LaY)_2_O_3_ samples vs. sintering temperature: 1—conventional heating, zero hold; 2—conventional heating, 10-h hold; 3—microwave heating, zero hold; 4—microwave heating, 10-h hold.

The average grain size was determined using the SEM images obtained on the polished surfaces of the samples. The microstructure of samples sintered under microwave and resistive heating at a temperature of 1750 °C with a 10-h isothermal hold is shown in Figure 8. Figure 9 shows the dependencies of the grain size (averaged across the diameter of the sample) on the sintering temperature at microwave and conventional heating for zero and 10-h hold times.

As seen from the data plotted in Figure 9, the heating method affects the grain growth rate substantially. At the same temperature and hold time, the average grain size obtained under microwave heating exceeds the grain size obtained under conventional heating greatly. For example, at a temperature of 1570 °C and zero hold the average grain size is 0.52 µm under conventional heating and 6.1 µm under microwave heating; at a temperature of 1750 °C and a 10-h hold the average grain size is 13.9 µm and 60.1 µm, respectively.

In the case of microwave heating, the character of the dependency of the average grain size on the temperature and hold time is typical of liquid phase sintering [38]. The rapid growth of the average grain size at intermediate temperatures (1550–1650 °C) corresponds to the predominant effect of the solution-reprecipitation mechanism at this stage. At higher temperatures the grain growth slows down and the microstructure coarsening by the Ostwald ripening mechanism can become the dominant process. This grain growth slowdown is as well characteristic of conventional and spark plasma liquid phase sintering [39,40] and it does not depend on the method of heating.

**Figure 10:**
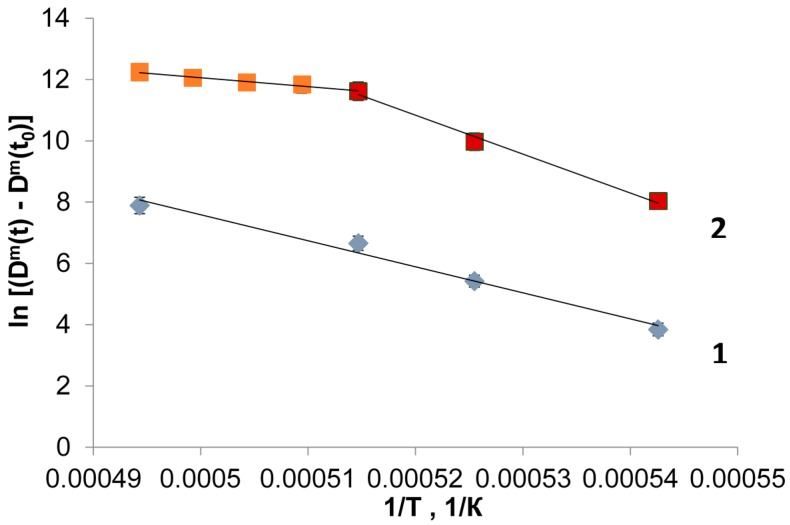
Plots of ln[(*D^m^*(*t*) − *D^m^*(*t*_0_)] vs. reciprocal temperature of sintering for conventional (**1**) and microwave (**2**) heating; here it is assumed that *m* = 3.

The activation energy of grain growth can be determined on the basis of the obtained data using Equation (5). Plotted in Figure 10 are the dependencies of the quantity ln[(*D^m^*(*t*) − *D^m^*(*t*_0_)] on the reciprocal temperature. The values of the activation energies and the coefficients of determination corresponding to a straight line fit of the Arrhenius dependence of ln[(*D^m^*(*t*) − *D^m^*(*t*_0_)] on *T*^−1^ are listed in Table 2 for different *m* values.

One or another mechanism controlling the grain growth could be chosen based on the comparison of the obtained values of the activation energy for grain growth with the known data on the activation energy for diffusion processes in the given material in the appropriate temperature range. Unfortunately, there are no data on the activation energy for diffusion available for the Y_2_O_3_—10 mol % La_2_O_3_ (doped with 5 at % Yb) solid solution.

It can be seen from the data listed in Table 2 that for the case of conventional heating the *R*^2^ values for both fits (*m* = 2 and *m* = 3) virtually do not differ. The hypothesis of *m* = 2 appears preferable because in this case the corresponding value of *Q_a_* is closer to the typical values of the activation energy of grain boundary diffusion of Y^3+^ ions in solids [41,42]. In the case of microwave heating, the plot of ln[(*D^m^*(*t*) − *D^m^*(*t*_0_)] shown in Figure 10 has two distinct parts for low (1570–1670 °C) and high temperatures (1670–1750 °C). This behavior is typical of liquid phase sintering due to the different mechanisms of grain growth acting at low and high temperature. The calculated values of the activation energy differ greatly for the two parts of the plot: for *m* = 3 the activation energy *Q_a_* = 241 kJ/mol for the temperature range 1570–1670 °C and *Q_a_* = 1056 kJ/mol for the range 1670–1750 °C. Large activation energy values for the high-temperature range are in good agreement with the activation energy for dissolution of rare-earth oxides which is above 1000 kJ/mol [43].

### 3.3. Ultra-Rapid Microwave Sintering of Al_2_O_3_, MgAl_2_O_4_, and Y_2_O_3_ Ceramics

The observed flash microwave sintering effect is not unique to Yb:(LaY)_2_O_3_ laser ceramics. The heating procedure, conditions and characteristic features of ultra-rapid microwave sintering have been studied for other oxide ceramics: Y_2_O_3_, Al_2_O_3_, and MgAl_2_O_4_ [18,44]. Similar to the case of Yb:(LaY)_2_O_3_ ceramics, the occurrence of the microwave flash sintering effect was in all cases determined by the combined action of two factors: the temperature of the sample and the microwave power deposited per unit volume of the sample, *P_v_*. The overheating of the samples was avoided and their integrity was ensured by a system of fast automatic process control, which prevented development of the thermal runaway. From the practical viewpoint, it is important that the onset of rapid densification is easily identified without dilatometry by a sharp decrease in the level of the input microwave power required to sustain the preset heating rate.

**Figure 11:**
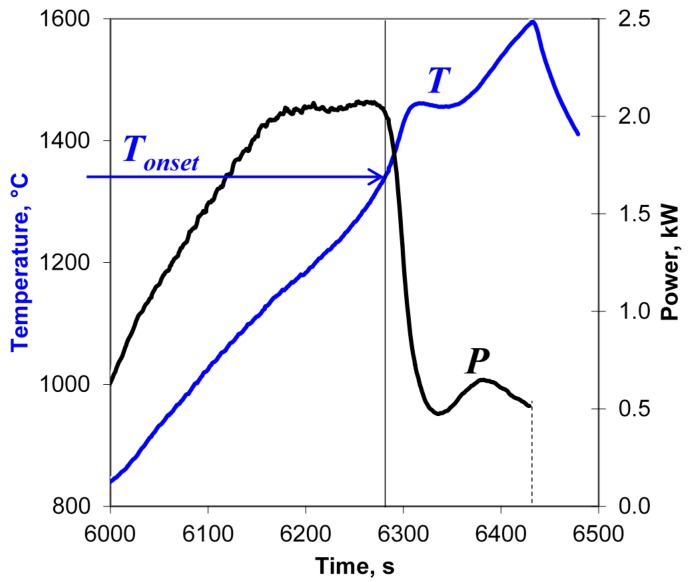
Temperature and microwave power at the applicator input vs. time, recorded at the high-temperature stage of the sintering of an Y_2_O_3_ sample with a heating rate of 100 °C/min. The vertical line denotes the onset of the sharp rise in the effective conductivity.

The Y_2_O_3_ samples were microwave heated at rates 10–200 °C/min to maximum temperatures 1400–1700 °C with zero hold time. Both a sharp drop in the input microwave power due to an increase in the material’s effective conductivity and a simultaneous increase in the density of the samples were observed at temperatures 1350–1450 °C when the microwave power deposited per unit volume of the sample, *P_v_*, was above 40 W/cm^3^. As an example, shown in Figure 11 are the temperature and the microwave power at the applicator input recorded at the high-temperature stage of the sintering of an Y_2_O_3_ sample with a heating rate of 100 °C/min to a maximum temperature of 1600 °C. Y_2_O_3_ samples with densities over 0.98 ρ_th_ have been obtained at a heating rate of 100 °C/min and maximum temperatures 1600–1700 °C. The density of the microwave flash sintered Y_2_O_3_ samples was higher than the density of the samples obtained by a slower-rate (10 °C/min) microwave heating process with a 15 min isothermal hold at the maximum temperature.

The Al_2_O_3_ powder compacts were microwave sintered in air at heating rates in the range 50–250 °C/min. The microwave power deposited per unit volume of the sample, *P_v_*, was 15–100 W/cm^3^. Samples, with the density as high as 98%–99% of the theoretical density (ρ_th_), were obtained at temperatures 1400–1550 °C with zero hold time. Additionally, Al_2_O_3_ samples were sintered in a preheated resistive furnace in the regimes which mimicked the regimes of microwave heating precisely, to make possible the comparison between the results of fast microwave and conventional sintering. The density of the samples sintered in the resistive furnace did not exceed 0.96 ρ_th_.

The density of the MgAl_2_O_4_ samples microwave heated at rates in the range 100–200 °C/min to a maximum temperature of 1800 °C with zero hold time was over 0.99 ρ_th_. This was markedly larger than the density of samples heated at a lower rate of 6 °C/min to the same temperature with a 2-h hold time (about 0.97 ρ_th_).

It should be noted that in contrast to Yb:(LaY)_2_O_3_-samples, the samples of all these compositions were compacted from high purity powders and contained no intentionally introduced additives, except for 0.05% MgO in Al_2_O_3_. Due to this fact, only minor amounts of liquid phase could be formed which were not clearly detectable by the analytical instruments used in this study. However, the specific behavior of the microwave power during the heating of the above listed ceramic materials, as well as the common features in the grain growth and microstructure formation suggest that their ultra-rapid densification at high values of the microwave power deposited per unit volume, *P_v_*, resulted from the same mechanism of fast mass transport via the softened particle surfaces as in the case of the Yb:(LaY)_2_O_3_ ceramic sintering.

### 3.4. Similarity between Microwave and DC/Low-Frequency AC Flash Sintering

The effect of flash sintering under an applied DC/AC voltage is usually discussed in terms of the electric field strength and the power absorbed per unit volume of the sample [4,7,29]. As was shown in [17], the observed microwave flash sintering effect can be discussed in similar terms.

The energy balance Equations (1) make it possible to estimate the microwave power absorbed per unit volume of the sample using the data on the rates of heating (*dT*/*dt*)_+_ and cooling (*dT*/*dt*)_−_ recorded in the experiments immediately before and after the microwave power switchoff. On variation of the microwave power input to the applicator from 0.5 to 3.0 kW, which corresponds to the variation of the heating rate from 50 to 2100 °C/min, the value of the power deposited per unit volume of the samples, *P_v_*, ranges from approximately 20 to 160 W/cm^3^. Note that the electric power density triggering the dc/ac flash sintering of various oxide ceramics is typically in the range 10–1000 W/cm^3^ [4,7,29]. From the relationship Equation (3) between the electromagnetic energy, *W*, stored in the cavity and the input power, *P*, taking into account the quality factor for the applicator used in the experiments, *Q* ≈ 10^4^, the microwave electric field strength can roughly be estimated as $E[\text{V}/\text{cm}]\approx 5\times \sqrt{P[\text{W}]}$. At the mentioned variation of the input power the microwave electric field strength in the samples varies from 100 to 270 V/cm. The electric field of the same order of magnitude is typically imposed on the samples in the experiments on DC/AC flash sintering.

Using Equation (2) it is possible to estimate the effective microwave electric conductivity, σ_eff_, based on the experimental data. It has been shown in [17] that the electric conductivity of the intergranular liquid phase can be estimated based on the percolation theory [45] assuming that it is much higher than the bulk conductivity of the grains. In particular, for the case when a sample was heated at a ramp-up rate of 2400 °C/min to 1500 °C it was estimated that the mass of the melted material could be as high as 20% of the total mass of the sample. The electric conductivity of the liquid phase was estimated to be 0.25–0.7 (Ω·cm)^−1^. The high-temperature electric conductivity of the 5 at % Yb^3+^ doped (La_0.1_Y_0.9_)_2_O_3_ composition is not known. However, it should be noted that the estimated values are slightly lower than the conductivity of pure Y_2_O_3_ above the melting point, which equals 0.9 (Ω·cm)^−1^ [46].

Thus, the values of the electric field strength and the power deposited per unit volume of the sample at which the flash sintering effect is observed are within the same ranges for the cases of microwave heating and the heating by DC/AC currents. The results of microstructure characterization of the sintered materials suggest that the mechanisms responsible for the flash sintering effect in the DC/AC electric field-assisted processes and microwave sintering are identical. The formation of isolated small, mostly rounded droplets located along the grain boundaries, similar to those described in Section 3.1, was observed in a study of the microstructure of BaCe_0.8_Gd_0.2_O_3-δ_ powder compacts heated conventionally under an applied ac electric voltage [6]. According to reference [6], the ac current flowing through the grain boundaries promotes grain welding via local Joule heating.

The fact that the flash sintering effect is perhaps more pronounced in the case of DC/AC field-assisted sintering could be associated with the significant difference in the geometrical configuration of the samples used in the experiments. This difference results in a different degree of temperature non-uniformity over the sample cross section. The samples used in the DC/AC flash sintering experiments were either dog bones with a cross section about 2 × 3 mm^2^ or small pellets, 5–7 mm in diameter, whereas the diameter of the pellets used in the microwave flash sintering experiments described here was 13 mm.

## 4. Conclusions and Outlook

We report the results of a study of an ultra-rapid (“flash”) microwave sintering process with oxide ceramic materials Al_2_O_3_, Y_2_O_3_, MgAl_2_O_4_, and Yb:(LaY)_2_O_3_ to densities 98%–99% of the theoretical value within minutes or even fractions of a minute, without the high-temperature hold stage. On the grounds of the analysis of experimental data (microwave power, heating and cooling rates) and microstructure characterization, we propose a mechanism of flash microwave sintering based on particle surface softening/melting.

At a certain point during rapid microwave heating of samples at a fixed rate (50, 100, 150, or 200 °C/min) a sharp drop of the input microwave power is observed. This drop in the input power is produced by the automatic process control system as a response to the rapid increase in the high-frequency electric conductivity, which is associated with the development of an overheating instability known as thermal runaway. The development of the instability depends on two factors: the temperature of the material and the microwave power deposited per unit volume. The onset temperature of the instability decreases with an increase in the microwave power deposited per unit volume of sample.

The results of the microstructure characterization of the sintered samples demonstrate that the ultra-rapid densification occurs due to particle surface softening and subsequent liquid phase sintering. The temperature of the softening/melting of particle surfaces/grain boundaries can be noticeably different from the bulk material melting point due to the elevated density of defects and impurities. During microwave volumetric heating the highest temperature arises in the core of the sample and the process of particle surface melting starts there. In the course of densification the liquid phase is extruded into a more porous peripheral structure and contributes to its densification. In effect, a densification front, coinciding with the region of the maximum deposition of the microwave power, propagates from the core of the sample to its periphery, producing a fully dense ceramic material in a very short time.

A comparison of the obtained results of microwave flash sintering with the results of DC or low-frequency AC flash sintering experiments suggests that the mechanisms of ultra-rapid densification are similar or even identical for the two approaches. The temperature distribution in the samples subjected to a DC/AC electric field is determined by volumetric Joule heating and the surface heat loss, being thereby similar to the microwave heating case. The estimates of the specific deposited power, electric field strength, and electric conductivity give similar values of these quantities for the cases of DC/AC and microwave flash sintering.

From the applications standpoint, it should be emphasized that an undeniable advantage of the microwave flash sintering process is the fact that no electrodes are needed to supply the power to the articles undergoing sintering. It is also important that in the case of microwave sintering the use of a relatively simple fast process control system can be instrumental in preventing possible damage resulting from thermal runaway.

Microwave flash sintering is a novel process that offers significant advantages over conventional sintering methods in terms of process duration and energy consumption. Its further development should be aimed at the study of such factors as the properties of powder materials, optimization of material composition, geometrical limitations (if any) imposed on the configuration of the sintered products, structural, functional properties, and performance of the materials obtained by microwave flash sintering.

## Figures and Tables

**Table 1:** Results of element analysis, wt %.

Element	Intragrain	Intergranular Phase
C	1.80 ± 0.09	1.50 ± 0.06
O	14.44 ± 0.47	17.36 ± 0.81
Y	68.83 ± 0.37	59.57 ± 2.00
La	9.13 ± 0.14	16.53 ± 1.70
Yb	5.79 ± 0.10	5.04 ± 0.18

**Table 2:** Activation energy of grain growth and the coefficient of determination, *R*^2^, for the dependence of ln[(*D^m^*(*t*) − *D^m^*(*t*_0_)] on the reciprocal temperature of sintering for microwave and conventional heating.

Method of Heating	Temperature (°C)	Activation Energy, *Q_a_* (kJ/mol)/ Coefficient of Determination, *R*^2^	
		*m* = 2	*m* = 3
microwave	1570–1670	723/0.992	1056/0.994
	1670–1750	147/0.9725	241/0.980
conventional	1570–1750	467/0.983	706/0.984
